# Systemic Therapies in Advanced Adrenocortical Carcinoma: Current Standards, Emerging Strategies, and Practical Considerations

**DOI:** 10.3390/cancers18203268

**Published:** 2026-10-10

**Authors:** Lina Pedraza Sanchez, Prerna Dogra, Elio Monsour, Guilherme Rabinowits

**Affiliations:** 1 Head and Neck-Endocrine Department, Moffitt Cancer Center, Tampa, FL 33612, USA; lina.pedrazasanchez@moffitt.org (L.P.S.);; 2 Morsani College of Medicine, University of South Florida, Tampa, FL 33620, USA

**Keywords:** adrenocortical carcinoma, systemic therapy, mitotane, cytotoxic chemotherapy, targeted therapy, immunotherapy

## Abstract

Adrenocortical carcinoma (ACC) is a rare endocrine malignancy characterized by marked biological heterogeneity and a high risk of recurrence and progression. While complete surgical resection remains the foundation of treatment for those with localized disease, systemic therapy is commonly used as adjuvant therapy for those with high-risk disease and for those whose ACC is not amenable to curative-intent surgery. Over the past decade, advances in molecular characterization have enhanced the understanding of ACC biology, leading to an increased number of systemic therapies tested for this disease. Unfortunately, the overall activity demonstrated has been low, and the optimal patient selection and treatment sequencing remain poorly defined. This review summarizes the evidence supporting the different systemic therapies currently in use for ACC and strategies for how best to select and sequence these treatment options in patients with advanced disease.

## Simple Summary

Adrenocortical carcinoma (ACC) is a rare and aggressive cancer with limited systemic therapy options. This review summarizes the available evidence for systemic therapies in advanced ACC, including chemotherapy, targeted therapies, immunotherapy, and emerging treatment combinations. We also propose a practical treatment algorithm to help clinicians select and sequence therapies according to disease characteristics and the available evidence, while highlighting current limitations and areas requiring further investigation.

## 1. Introduction

Adrenocortical carcinoma (ACC) is a rare and aggressive endocrine malignancy, with an annual incidence of approximately 1–2 per million in the United States [1]. At diagnosis, approximately 40–65% of patients have advanced (here defined as locally advanced, not amenable to curative-intent surgery, or metastatic) ACC (European Network for the Study of Adrenal Tumors [ENSAT] stage III–IV). While locally advanced disease may still be amenable to curative-intent surgical resection, stage IV disease remains largely incurable, with systemic therapy being the mainstay of treatment [2,3].

ACC is characterized by clinical, molecular, and pathological heterogeneity. Up to two-thirds of tumors are hormonally active, with cortisol excess being the most common presentation. At the molecular level, IGF2 overexpression is reported in most ACCs, and the most frequently disrupted pathways include p53/Rb1 and Wnt/β-catenin signaling [4,5,6]. Integrated multi-omics profiling has stratified ACC into three molecular subtypes (COC1, COC2, COC3) with markedly different prognoses, but these classifications have not yet been validated as predictive biomarkers for systemic therapy selection [7]. ACC generally exhibits an immunologically “cold” tumor microenvironment (TME) with low levels of tumor-infiltrating lymphocytes (TILs), particularly in cortisol-secreting tumors, which are associated with unfavorable outcomes [8]. Traditional biomarkers such as programmed death-ligand 1 (PD-L1) expression and defective mismatch repair (dMMR) are detected in a minority of tumors and have not been shown to reliably predict response to immune checkpoint inhibitors (ICIs) in ACC [9,10,11,12].

While 5-year overall survival (OS) for stage IV disease is approximately 10–15%, emerging evidence suggests a trend toward improving outcomes. This trend may reflect, in part, care at experienced centers and more individualized use of available treatments (Table 1) [13,14,15]. In this review, we provide a comprehensive overview of systemic therapy for advanced ACC, discussing the supporting evidence for the current treatment approaches and highlighting emerging therapeutic strategies.

## 2. Mitotane Monotherapy

Mitotane is the only FDA-approved drug in ACC. Etoposide, doxorubicin, cisplatin, and mitotane (EDP-M) combination remains the preferred first-line regimen for patients with advanced ACC (further details are provided below in the cytotoxic chemotherapy section). Mitotane monotherapy may be considered in carefully selected patients with low-volume, slowly progressive disease and minimal or no symptoms, although this approach is supported primarily by retrospective data and has not been compared prospectively with EDP-M [16,17].

In the largest published cohort of mitotane monotherapy in advanced ACC (n = 127), the overall response rate (ORR) was 20.5%, with a median progression-free survival (PFS) of 4.1 months (range: 1–73) and median OS of 18.5 months (range: 1.3–220) [17]. On multivariate analysis, two independent predictive factors for clinical benefit were identified: low tumor burden (<10 metastatic lesions) and disease-free interval (DFI) greater than one year after initial curative-intent therapy. Sixty-seven percent of patients with both favorable features achieved a clinical benefit, with stable disease greater than 180 days. Furthermore, patients who achieved therapeutic mitotane plasma concentrations higher than 14 mg/L had significantly longer OS (HR 0.42; *p* = 0.003) [17].

More recently, Mangone et al. developed the ENSAT Risk Score for Advanced ACC in a multicenter international cohort of 418 patients receiving systemic therapy [18]. This score integrates tumor burden, cortisol excess, performance status, and neutrophil-to-lymphocyte ratio. A score > 2 (poor-risk) was significantly associated with worse OS and time to tumor progression (TTP) across all treatment groups and predicted poorer response to mitotane monotherapy (*p* < 0.01) and second-line therapies (*p* = 0.04), providing a practical tool for selecting patients most likely to benefit from systemic therapy [18].

**Figure 1:**
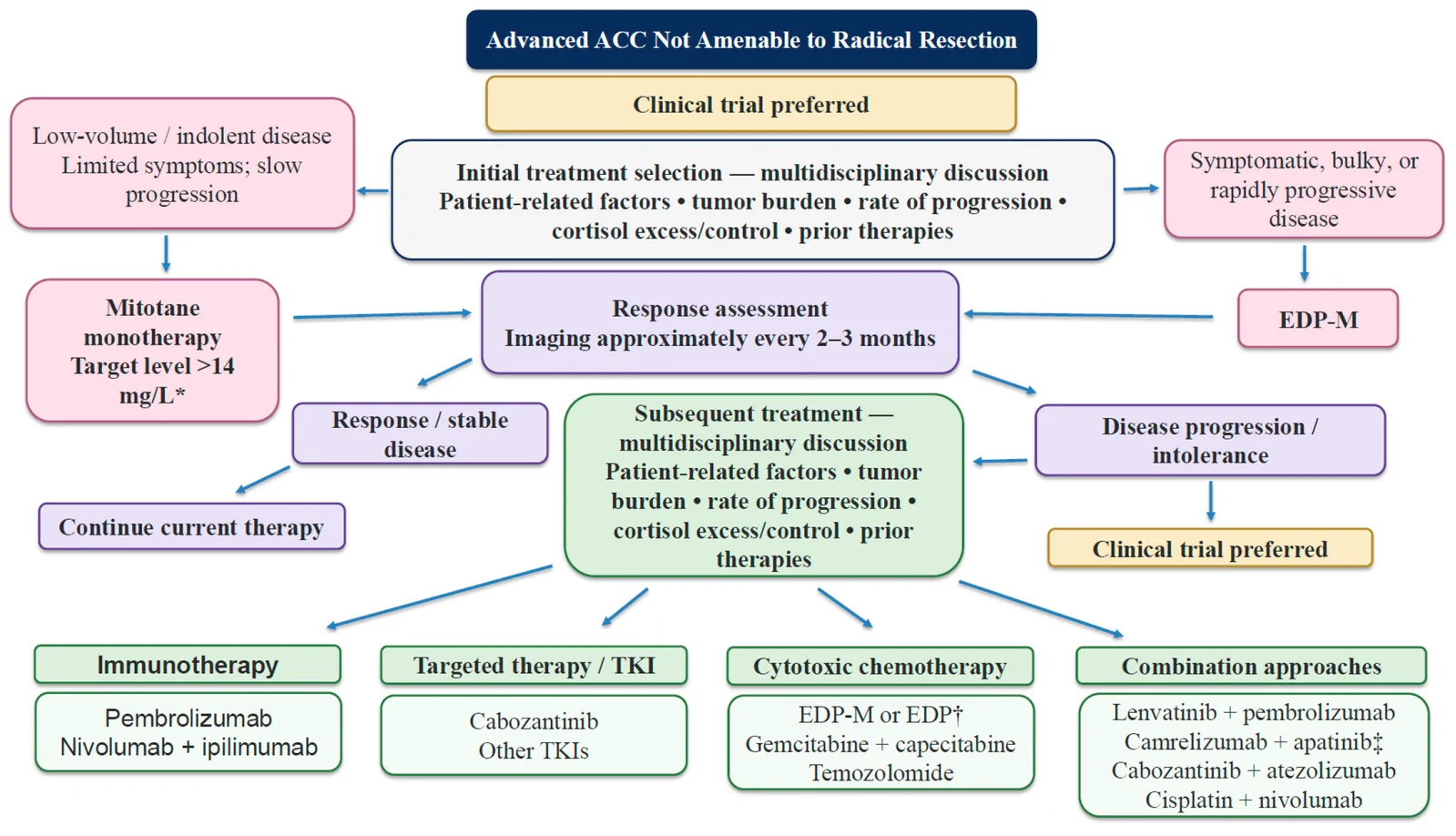
Practical treatment framework for systemic therapy in advanced ACC. Mitotane monotherapy is an option only for carefully selected patients; the combination approaches shown are investigational and preferably used in clinical trials. Other subsequent-line approaches differ in strength of evidence and should not be interpreted as equivalent options. Treatment selection should be individualized and discussed in a multidisciplinary setting; clinical trial enrollment should be considered before first-line therapy and at each subsequent decision point. * Therapeutic mitotane target generally > 14 mg/L † EDP may be considered as subsequent cytotoxic therapy, particularly when mitotane is not appropriate or is being discontinued. ‡ Neither camrelizumab nor apatinib is FDA-approved in the United States. Abbreviations: ACC, adrenocortical carcinoma; EDP, etoposide, doxorubicin, cisplatin; EDP-M, EDP plus mitotane; FDA, U.S. Food and Drug Administration; TKI, tyrosine kinase inhibitor.

In a population-based study from the Netherlands involving 167 patients with metastatic ACC, patients treated with single-agent mitotane had a 5-year OS of 11.4%; EDP-M was not associated with a statistically significant improvement in OS compared with other treatments (11.8 vs. 5.6 months; *p* = 0.525) [19]. These findings may have been confounded by differences in baseline disease characteristics and treatment selection and should not be interpreted as evidence that single-agent mitotane and EDP-M have equivalent efficacy. Mitotane monotherapy may be considered for selected patients with low-volume, slowly progressive disease and prolonged DFI, whereas EDP-M is generally preferred when more rapid tumor control is needed, such as in patients with bulky, symptomatic, or rapidly progressive disease (Figure 1) [16,17,18,20].

Despite this evidence of activity in selected patients, mitotane efficacy in practice is also influenced by substantial pharmacologic and tolerability challenges. Achieving therapeutic levels can be limited by its toxicity profile; heterogeneous tumor expression of enzymes that metabolize mitotane; individual body habitus and fat deposition of mitotane, leading to its variable half-life; and multiple drug–drug interactions that may also interfere with its metabolism and activity. Future studies should focus on identifying those patients who are more likely to benefit from mitotane and on optimizing individualized dosing and monitoring strategies.

In clinical practice, mitotane concentrations are generally targeted to 14–20 mg/L and monitored regularly during dose escalation and maintenance, alongside tolerability and endocrine function [16,21]. Mitotane induces CYP3A4, and the interaction can persist after treatment is stopped; medication lists should be reviewed when starting or stopping mitotane, particularly for drugs with exposure affected by CYP3A4 induction. Most patients on long-term mitotane require glucocorticoid replacement, often at doses higher than those used for conventional adrenal insufficiency because mitotane increases cortisol-binding globulin and accelerates steroid clearance. Mineralocorticoid replacement may also be needed. For cortisol-secreting ACC, clinically significant hypercortisolism should be managed promptly; metyrapone or osilodrostat may be considered when rapid biochemical control is required. Total serum cortisol can be misleading during mitotane therapy, and endocrine input is valuable when adjusting replacement or treating cortisol excess [16,21].

## 3. Cytotoxic Chemotherapy

As discussed above, for patients with advanced or rapidly progressive disease, cytotoxic chemotherapy with or without mitotane is considered the cornerstone of systemic treatment. Treatment selection is guided by disease burden, rate of progression, symptoms, performance status, and prior systemic treatments [21].

### 3.1. Etoposide, Doxorubicin, Cisplatin and Mitotane (EDP-M)

A prospective, phase 2, multicenter study evaluated the activity of EDP-M in 72 patients with ACC not amenable to radical surgery [22]. The ORR was 48.6% (95% CI: 37.1–60.3%), with 5 patients (7%) achieving a complete response. Ten patients underwent radical surgical resection for residual disease after chemotherapy. Median TTP and OS for the entire cohort were 9.1 and 28.5 months, respectively, and 18.2 and 47.7 months, respectively, among those attaining a disease response [22].

Building on these promising results, the first international randomized trial in locally advanced and metastatic ACC (FIRM-ACT) was designed [20]. This phase III randomized trial compared EDP-M (n = 151) with streptozocin and mitotane (n = 153) in patients with advanced ACC. EDP-M demonstrated a higher ORR (23.2% vs. 9.2%; *p* < 0.001), disease control rate (DCR) (58.3% vs. 31.4%; *p* < 0.001), and median PFS (5.0 months vs. 2.1 months; *p* < 0.001). Although OS was numerically higher with EDP-M (14.8 vs. 12 months; *p* < 0.07), this difference was not statistically significant, likely because of the trial’s crossover design. There was a trend toward increased OS among the 54 patients whose blood mitotane level was 14 mg/L or higher compared with the 212 patients whose levels were lower than 14 mg/L (HR, 0.76; 95% CI, 0.54 to 1.08; *p* = 0.13). Additionally, approximately 10% of patients died of causes unrelated to disease progression, including treatment-related complications. It is important to note that the activity of these regimens was retained when administered after crossover. However, FIRM-ACT was not designed to establish the optimal treatment sequence, so the crossover data do not establish equivalent efficacy between these sequences or justify routinely deferring EDP-M [20].

Based on these results, EDP-M is considered the standard first-line regimen for patients with advanced ACC.

### 3.2. Streptozotocin Plus Mitotane

A phase 2 prospective study evaluated streptozocin in combination with mitotane in 40 patients with localized disease (n = 25) and with metastasis (n = 15) at the time of diagnosis [23]. Of the 28 radically operated patients, 17 received streptozocin in the adjuvant setting, and 11 received no adjuvant therapy post-surgery. The median DFI for those who underwent curative-intent surgery was longer for those who received adjuvant streptozocin (49 months), in comparison to those who waited until developing recurrent or metastatic disease (12 months). Among the 12 patients who presented with advanced disease at diagnosis, the ORR was 36.4%, and the median OS was 16 months [23].

Based on these results, streptozotocin plus mitotane was chosen as the comparator arm in the above-reported FIRM-ACT trial, demonstrating lower activity in comparison to EDP-M [20].

### 3.3. Gemcitabine and Fluoropyrimidine-Based Therapy

There is no established standard second-line therapy for advanced ACC. Gemcitabine plus fluoropyrimidine-based regimens have demonstrated modest activity in previously treated patients. In a prospective single-arm phase 2 study, 28 patients with advanced ACC who had received one or two prior lines of therapy received gemcitabine combined with 5-fluorouracil (or capecitabine) and ongoing mitotane [24]. The study reported an ORR of 7.1%, a 4-month DCR of 46.4% (95% CI: 27.6–65.2), TTP of 5.3 months (range: 1–43 months), and OS of 9.8 months (range: 3–73 months). Serum mitotane levels were monitored in 19 patients. Those whose mitotane levels were above the therapeutic threshold (14 mg/L) had a longer TTP than patients with lower mitotane levels (*p* = 0.13). Three of the 14 patients with hormone-secreting ACC who had stable disease on CT scans achieved a partial hormone response and significant clinical improvement. Treatment was well tolerated, with grade 3 and 4 toxicities consisting of leukopenia (21.4%), thrombocytopenia (3.5%), and mucositis (3.5%) [24].

In a larger retrospective analysis of German and Italian series, 30 (20.8%) of 132 patients with advanced ACC treated with gemcitabine and capecitabine experienced a clinical benefit from treatment for at least 4 months, with a median duration of 33.6 weeks (range: 17–94 weeks). Twenty-seven of these 30 patients received concomitant mitotane. Those who reached a mitotane level greater than 14 mg/L had a longer PFS than those who did not reach that level or did not receive mitotane (HR for progression, 0.56; 95% CI, 0.35 to 0.77; *p* = 0.0026) [25].

In another retrospective study of 50 patients with advanced ACC treated with a gemcitabine and capecitabine combination, the 4-month DCR was 30%, with a median PFS of 3.0 months and disease-specific survival (DSS) of 8.0 months [26].

These data suggest that gemcitabine-based regimens may provide temporary disease control in some patients, particularly those who were able to achieve therapeutic mitotane levels, although objective responses are uncommon.

### 3.4. Temozolomide

A multicenter retrospective study with 28 patients with advanced ACC, treated in second- to fourth-line therapy with temozolomide, reported an ORR of 21.4%, DCR of 35.8%, median PFS of 3.5 months, and OS of 7.2 months. Treatment was well tolerated, and disease response was more common in patients with methylation of the O6-methylguanine-DNA methyltransferase (MGMT) gene [27].

Most recently, a real-world experience with temozolomide in advanced ACC was reported in abstract form. Nine patients (28.1%) received temozolomide in the second line, 12 (37.5%) in the third line, and 11 (34.4%) in the fourth and fifth lines. Thirty patients (93.8%) received concurrent mitotane, and the therapeutic concentration was achieved in the previous treatment line in 81.3% (n = 26). The activity was lower than previously reported (likely due to this patient population), with a single patient (3.1%) achieving a partial response and 11 (34.4%) stable disease, for a 6-month or greater DCR of 21.9% (n = 7). The median PFS was 1.9 months (95% CI 1.87–1.93), and the median OS was 8.2 months (95% CI 3.3–13.2). Grade 3 and 4 toxicities were observed in 18.8% (n = 6) of the patients [28].

### 3.5. Cabazitaxel

Cabazitaxel, a second-generation taxane, showed limited activity when given as second- or third-line therapy in advanced ACC based on a prospective phase II trial of 25 patients. No responses were seen, but the 4-month DCR was 24%, with a median PFS of 1.5 months and a median OS of 6.0 months [29].

Overall, cytotoxic chemotherapy has modest activity in ACC. EDP-M remains the preferred cytotoxic regimen, supported by a prospective randomized phase III trial for patients with advanced ACC. Nevertheless, given its toxicity, initial mitotane monotherapy may be considered in carefully selected patients with indolent, low-volume, and asymptomatic disease, after discussion of the uncertain sequencing evidence. Finally, clinical trial enrollment, if available, should always be the preferred option for these patients given the overall limited activity of currently acceptable regimens.

## 4. Targeted Therapy

### 4.1. IGF Pathway Inhibition

IGF-2 is overexpressed in ~85–90% of ACC due to epigenetic alterations at the 11p15.5 locus, driving proliferation through IGF-1R and insulin receptor isoform A signaling [4,30]. Despite this, clinical outcomes with therapies targeting this receptor have been disappointing. The phase III randomized, double blind, placebo-controlled GALACCTIC trial evaluated linsitinib (a dual IGF-1R/insulin receptor inhibitor) versus placebo (2:1 randomization) in 139 patients with advanced ACC treated in the second- or third-line setting. The study was unblinded early for futility, with no improvement in PFS or OS and only a 3% response rate [31].

Similarly, the anti-IGF-1R antibody cixutumumab in combination with the mTOR inhibitor temsirolimus demonstrated stable disease greater than 6 months in 42% of the 26 treated patients but no objective responses. The lack of activity of these agents likely reflects redundancy within the IGF signaling network and compensatory growth factor pathways [32].

### 4.2. Anti-Angiogenic Tyrosine Kinase Inhibitors (TKIs)

Given the limited efficacy of conventional cytotoxic chemotherapy and the molecular complexity of ACC, multiple targeted therapies have been evaluated, particularly the anti-angiogenic TKIs. VEGF is overexpressed in 71–86% of ACCs compared to only 17–25% of adrenal adenomas, and 0% in the normal adrenal cortex, suggesting a role in the development of ACC and the rationale for the use of anti-angiogenic TKIs [33].

#### 4.2.1. Cabozantinib

Cabozantinib, a multi-TKI targeting VEGF, c-MET, AXL, and RET receptors, has demonstrated promising activity in ACC.

A retrospective study of 16 patients with advanced ACC treated with cabozantinib reported a median PFS of 16 weeks (3–61) and an OS of 58 weeks (6–83). Three (19%) patients had a partial response and five (31%) had stable disease [34].

In a prospective, phase II study of 18 heavily pretreated patients with a median of 2 prior lines of therapy (range 0–5 or more), cabozantinib achieved a median PFS of 6 months (95% CI 4.3 to not reached) and a median OS of 24 months (95% CI 15.6 months to not reached) [35]. Disease control was observed in 78% (95% CI 52–94%) of the patients, although objective responses were low at 11·1% (95% CI 1·4–35%). Patients who had received mitotane within 6 months of cabozantinib dosing start had a shorter treatment duration than those who stopped mitotane more than 6 months prior to study initiation. Among the 10 patients with cortisol-producing tumors with restaging scans on study, two (20%) had disease progression, seven (70%) had stable disease, and one patient had a confirmed partial response. Treatment-related adverse events (TRAEs) of grade 3 or higher occurred in 11 (61%) of 18 patients [35]. Although exciting, it is unclear if the prolonged median PFS and OS seen in this study were due to treatment effect or patient selection and disease biology; the median time between the ACC diagnosis and the start of cabozantinib therapy was 23.8 months (IQR 12.4–46.2).

It is important to emphasize the interaction between mitotane and cabozantinib. Since mitotane is a potent CYP3A4 inducer, concurrent therapy can significantly reduce cabozantinib plasma concentrations and compromise its efficacy. Furthermore, this effect is not specific to cabozantinib. As another example, Kroiss and colleagues [36] studied the effect of another anti-angiogenic TKI (sunitinib) in 38 patients with refractory ACC. Sunitinib was associated with a median PFS of 83 days (95% CI 80–85), a median OS of 5.4 months (3.2–7.6), and five patients (15%) had stable disease as their best response. This low treatment activity was mostly attributed to prior mitotane use. Because mitotane elimination and CYP3A4 induction vary among patients, no fixed washout interval has been validated. Although serial mitotane levels and a review of the specific anti-angiogenic TKIs’ interaction profile may help guide treatment timing, low to undetectable mitotane levels may not reliably predict CYP3A4 activity over time because of mitotane’s fat deposition and prolonged, variable half-life [37].

#### 4.2.2. Axitinib

Axitinib, an oral anti-angiogenic multi-TKI of VEGF, PDGF, and c-KIT receptors, was tested in a prospective phase II study with 13 pre-treated patients with advanced ACC. Dose escalation was permitted if the administered dose was tolerable. No objective responses were seen, and 10 of 13 patients experienced grade 3 and 4 toxicities. The median PFS and OS were 5.5 and 13.7 months, respectively [38].

#### 4.2.3. Lenvatinib

A systematic review and single-arm meta-analysis of 11 studies (208 patients) evaluating different TKIs, including lenvatinib, either as monotherapy or in combination with immunotherapy in advanced ACC reported an ORR of 15% and a median OS range of 5.4–26.9 months in the monotherapy group (n = 93; 5 studies) and an ORR of 26% and a median OS range of 13.5–30.6 months in the combination group (n = 118; 6 studies), providing the rationale for a prospective study. Although these findings suggest increased activity with combination therapy, these heterogeneous, nonrandomized cohorts cannot establish a comparative benefit or a synergistic effect [39].

## 5. Immunotherapy

The rationale for immunotherapy is supported by the ACC immune microenvironment, which demonstrates PD-L1 expression in 10–20% of tumor cells and in approximately 70% of tumor-infiltrating mononuclear cells, and PD-L2 expression in 44% of ACC samples [9,10]. Despite that, ICIs have shown modest activity in advanced ACC, with durable responses observed in only a minority of patients [11,40].

### 5.1. Pembrolizumab

Pembrolizumab targets programmed cell death protein 1 (PD-1) and has the most robust prospective data in ACC.

In an investigator initiated, single center, phase II study, including 39 patients with advanced ACC (31% received more than 1 prior line of treatment), pembrolizumab demonstrated an ORR of 23% (95% CI, 11–39%) (all partial responses), DCR of 52% (95% CI, 33–69%), median PFS of 2.1 months (95% CI, 2.0–10.7 months), and median OS of 24.9 months (95% CI, 4.2 months to not reached). Treatment was overall well tolerated, with grade 3 and 4 TRAEs seen in 5 patients. There was no significant association between treatment response and tumor PD-L1 status, tumor mutation burden (TMB), TIL score, or somatic alterations. Interestingly, all patients with an objective response had low- to moderate-volume disease, and the authors suggested that these responders may have had a more indolent growth pattern [11].

In another investigator-initiated, single-center, open-label phase II study of pembrolizumab for multiple rare cancers, regardless of PD-L1 expression, 23 patients with advanced ACC were enrolled, including 4 cases of cortisol-producing tumors (17%). The median number of prior lines of systemic therapy before pembrolizumab administration was two (range, 1–5). Among the 20 evaluable patients, the ORR was 20% (all partial responses), the median DOR was 17.6 months, the median PFS was 4.0 months (95% CI, 2.0–6.2 months), and the median OS was 15.5 months (95% CI, 6.2–22.8 months). Thirty percent of patients were alive without progression at 27 weeks (the primary endpoint of this study). Treatment was well tolerated, with three grade 3 or higher TRAEs, including increased liver enzyme levels and pneumonitis. PD-L1 expression did not correlate with advanced disease stage, high tumor grade, or OS [40].

Other anti-PD(L)1 agents like nivolumab and avelumab have also been tested, demonstrating limited activity in patients with advanced ACC [41,42].

### 5.2. Nivolumab Plus Ipilimumab

In an attempt to improve the limited activity seen with single-agent ICI, combined immunotherapy with nivolumab plus ipilimumab (anti-cytotoxic T lymphocyte associated protein 4 [CTLA-4]) was evaluated in 21 patients with advanced ACC as part of a prospective phase II basket cohort of rare cancers trial (SWOG S1609). The median number of prior lines of therapy was 2 (range: 1–9). Three (14%) patients had confirmed partial response; one had stable disease greater than 6 months for a CBR of 24%. The median PFS and OS were 1.8 months and 15.8 months, respectively, and grade 3 and 4 TRAEs occurred in 52% of patients [43].

Predictive biomarkers for immunotherapy remain poorly defined in ACC. Altieri et al. reported dMMR in 14% of 109 ACCs and MSI-H in approximately 1% [12]. These findings cannot be generalized as the prevalence in unselected ACC; discordance between assays has been reported, and immunotherapy responses in ACC were not restricted to these tumors [11,12]. Likewise, PD-L1 expression and TMB have not been validated as reliable predictive biomarkers [11,44]. In addition, cortisol excess may negatively affect immunotherapy efficacy because of its immunosuppressive effects, although its predictive value in ACC has not been established [8]. At the molecular level, these observations are further supported by recent analyses of steroid phenotypes. Steroid phenotype stratification of the TCGA-ACC cohort demonstrated that tumors with a high steroid-production phenotype were associated with suppressed HLA expression and a lymphocyte-depleted “cold” TME, while those with a low steroid-production phenotype displayed elevated HLA expression, enriched T-cell infiltration, and upregulation of immune checkpoints (PDCD1, CTLA4), consistent with an inflamed but exhausted TME; however, these findings are exploratory [45]. No ACC-specific biomarker has been validated to reliably select patients for immunotherapy. Control of clinically significant hypercortisolism before and during ICI is a reasonable clinical consideration, both to address the complications of cortisol excess and its immunosuppressive effects. Whether pharmacologic cortisol control improves ICI efficacy has not been established prospectively, and cortisol secretion alone should not be used as a validated predictive biomarker [21,44].

## 6. Combination Agents

### 6.1. Lenvatinib Plus Pembrolizumab

Bedrose et al. reported the results of lenvatinib and pembrolizumab combination as salvage therapy in 8 patients (3 hormonally active) with advanced ACC that progressed after a median of 4 (range: 2–9) prior lines of therapy [46]. Interestingly, 6 (75%) patients had disease progression on prior ICI and 5 (62.5%) had progressed on prior TKI therapy. The median PFS was 5.5 months (95% CI 1.8–not reached) and the median duration of therapy was 8.5 months (range: 2–22). Two (25%) patients had a partial response, and one (12.5%) had stable disease. This small retrospective study requires confirmation; a prospective phase 2 trial testing this combination in advanced ACC is ongoing (ACCOMPLISH trial - NCT05036434) [47].

### 6.2. Camrelizumab Plus Apatinib

Camrelizumab (anti-PD-1) plus apatinib (anti-VEGFR) demonstrated encouraging activity in a small, single-arm phase II study of advanced ACC. This study enrolled 21 patients with advanced ACC that failed prior lines of therapy. The study reported an ORR of 52% (95% CI 30–74%) and a DCR of 95%, with median PFS and OS of 13.3 and 20.9 months, respectively. Exploratory biomarker analyses suggested that patients with higher peripheral CXCR3^+^ CD8^+^ T cells and greater overlap between TILs and circulating T-cell clonotypes were more likely to respond to therapy [48].

### 6.3. Cabozantinib Plus Atezolizumab

A cohort of 24 patients with advanced ACC was enrolled in the prospective, phase II, multicenter CABATEN trial evaluating the antiangiogenic TKI cabozantinib with the anti-PD-L1 agent atezolizumab. The ORR was 8.3% (95% CI: 1–27), the duration of response was 13.1 months, the median PFS was 2.9 months (95% CI: 2.8–5.7), and the median OS was 13.5 months (95% CI: 8.8-NR) [49].

### 6.4. Cisplatin Plus Nivolumab

A proposed rationale for this approach is that cisplatin may enhance ICI activity through induction of cell death and increased tumor antigen presentation. In a retrospective analysis of 23 patients with advanced ACC who had progressed on EDP-M with or without additional prior regimens, the combination of cisplatin plus nivolumab demonstrated an ORR of 34.8% (all partial responses), a 6-month DCR of 39.1% and an overall CBR of 52.2%. The median PFS was 4.3 months, and the median OS was 18.9 months. Treatment was generally well tolerated, with most adverse events limited to grade 1 and 2 toxicities; only one patient discontinued therapy because of grade 3 ICI-related hepatitis [50]. This regimen remains investigational pending prospective validation.

### 6.5. Mitotane Plus Pembrolizumab

The rationale for combining mitotane with an ICI is supported by two proposed, unproven mechanisms: early mitotane-induced suppression of tumor cortisol production may reduce glucocorticoid-mediated immunosuppression within the tumor microenvironment, while sustained mitotane exposure at therapeutic levels promotes adrenolytic cell death that may release tumor-specific antigens and further enhance ICI activity [8,16]. In a retrospective review of 6 patients with metastatic ACC who had failed mitotane with or without chemotherapy and received pembrolizumab with ongoing mitotane therapy, all 6 patients were alive at least 16 months after starting the novel combination, with responses observed in both microsatellite instability-high and microsatellite-stable tumors [51]. Based on this signal, a prospective phase II trial of pembrolizumab plus mitotane in advanced ACC (NCT05634577) was initiated but was ultimately terminated due to slow accrual, underscoring the persistent challenge of enrolling adequately powered combination trials in this rare disease.

Across combination studies, the evidence remains limited by small cohorts, single-arm designs, and, for several regimens, retrospective patient selection. These regimens are investigational and should preferably be studied in clinical trials rather than presented as established alternatives to EDP-M. Molecular profiling in patients with incurable ACC may help identify actionable alterations and support enrollment in biomarker-directed studies [21,46,48,49,50,51].

## 7. Emerging Therapies

### 7.1. Antibody-Drug Conjugates (ADCs)

A first-in-human phase I trial (NCT06041516) is currently evaluating ADCT-701, an antibody-drug conjugate targeting Delta-like non-canonical Notch ligand 1 (DLK1), in patients with advanced neuroendocrine neoplasms. DLK1 is highly expressed in ACC, and preclinical studies demonstrated that ADCT-701 suppresses tumor growth and improves survival in DLK1-expressing models. This trial represents a novel biomarker-driven targeted therapy approach in ACC, and results are eagerly awaited [52,53].

### 7.2. Radiopharmaceuticals

Emerging theranostic and radiopharmaceutical strategies are being investigated for patients with ACC progressing after standard therapies. In a small study of 13 patients treated with [131I]IMAZA, which targets 11-beta-hydroxylase, five patients whose disease stabilized had a median PFS of 14.3 months (range 8.3–21.9) and limited toxicity. Other investigational targets include CXCR4, somatostatin receptors, and PSMA, although clinical experience remains limited [54,55].

In a French retrospective study, 27 patients with ACC participated in 30 early-phase clinical trials, predominantly after prior systemic therapy. Patients had received a median of 2 prior lines of therapy. Despite this heavily pretreated population, the DCR with the investigational therapy was 61%, with 52% of evaluable patients achieving a longer PFS when compared with their preceding systemic therapy [56]. These results are subject to bias but support clinical trial enrollment.

**Table 1:** Selected systemic therapies for advanced adrenocortical carcinoma and key clinical outcomes.

Therapy Category	Regimen/Drug(s)	First Author (Year)	Study Design	Patients (N)	Key Outcomes	Treatment Setting and Practical Notes
Mitotane	Mitotane monotherapy	Megerle (2018) [17]	Multicenter retrospective cohort	127	ORR 20.5%; median PFS 4.1 mo; median OS 18.5 mo	Selected low-volume/indolent disease; initial treatment
Cytotoxic chemotherapy	EDP-M	Fassnacht (2012) [20]	Prospective, phase III RCT (FIRM-ACT)	151	ORR 23.2%; median PFS 5.0 mo; median OS 14.8 mo	Preferred first-line for symptomatic/bulky/rapidly progressive disease
Cytotoxic chemotherapy	Streptozotocin + mitotane	Fassnacht (2012) [20]	Prospective, phase III RCT comparator arm (FIRM- ACT)	153	ORR 9.2%; median PFS 2.1 mo; median OS 12.0 mo	First-line comparator in FIRM-ACT; lower activity than EDP-M
Cytotoxic chemotherapy	Gemcitabine + 5-FU/capecitabine ± mitotane	Sperone (2010) [24]	Prospective, phase II, single-arm	28	ORR 7.1%; 4-mo DCR 46.4%; median TTP 5.3 mo; median OS 9.8 mo	Second or third-line option; limited activity
Cytotoxic chemotherapy	Temozolomide	Cosentini (2019) [27]	Multicenter, retrospective	28	ORR 21.4%; DCR 35.8%; median PFS 3.5 mo; median OS 7.2 mo	Second- to fourth-line
Cytotoxic chemotherapy	Cabazitaxel	Laganà (2022) [29]	Prospective phase II, single-arm	25	ORR 0%; 4-mo DCR 24%; median PFS 1.5 mo; median OS 6.0 mo	Second or third line; limited activity
Targeted therapy	Cabozantinib	Campbell (2024) [35]	Prospective phase II, single arm	18	ORR 11.1%; median PFS 6.0 mo; median OS 24.0 mo	Predominantly previously treated; monitor prior mitotane exposure (CYP3A4 interaction)
Targeted therapy	Axitinib	O’Sullivan (2014) [38]	Prospective phase II, single arm	13	ORR 0%; median PFS 5.48 mo; median OS > 13.7 mo	Previously treated; limited activity
Targeted therapy	Linsitinib	Fassnacht (2015) [31]	Prospective phase III RCT	139	ORR 3%; no PFS or OS benefit vs. placebo	Second or third line; trial stopped early for futility.
Immunotherapy	Pembrolizumab	Raj (2020) [11]	Prospective phase II, single-arm	39	ORR 23%; DCR 52%; median PFS 2.1 mo; median OS 24.9 mo	Mixed prior exposure
Immunotherapy	Pembrolizumab	Chahla (2025) [40]	Prospective phase II, single arm	20	ORR 20%; CBR 30% at 27 wks; median PFS 4.0 mo; median OS 15.5 mo	Previously treated
Immunotherapy	Nivolumab + ipilimumab	Patel (2024) [43]	Prospective phase II basket trial, single arm	21	ORR 14%; CBR 24%; median PFS 1.8 mo; median OS 15.8 mo	Previously treated; TRAE-related discontinuation 21%
Combined therapy	Lenvatinib + pembrolizumab	Bedrose (2020) [46]	Retrospective case series	8	ORR 25%; median PFS 5.5 mo	Previously treated; investigational; prospective ACCOMPLISH trial is ongoing
Combined therapy	Camrelizumab + apatinib	Zhu (2024) [48]	Prospective phase II, single arm	21	ORR 52%; DCR 95%; median PFS 13.3 mo; median OS 20.9 mo	Previously treated; neither agent is FDA-approved in the United States
Combined therapy	Cabozantinib + atezolizumab	Capdevila (2025) [49]	Prospective phase II basket trial, single arm	24	ORR 8.3%; DCR 33.3%; median PFS 2.9 mo; median OS 13.5 mo	Previously treated; investigational
Combined therapy	Cisplatin + nivolumab	Laganà (2026) [50]	Retrospective, single-center cohort	23	ORR 34.8%; 6-mo DCR 39.1%; median PFS 4.3 mo; median OS 18.9 mo	After EDP-M; investigational

Note. Selected studies are shown to summarize clinically relevant evidence. Abbreviations: ACC, adrenocortical carcinoma; AE, adverse event; CBR, clinical benefit rate; DCR, disease control rate; ORR, objective response rate; OS, overall survival; PFS, progression-free survival; PR, partial response; mo, months; wks, weeks; RCT, randomized controlled trial; SD, stable disease; TRAE, treatment-related adverse event; TTP, time to tumor progression; EDP-M, etoposide, doxorubicin, cisplatin plus mitotane; FDA, U.S. Food and Drug Administration; 5-FU, 5-fluorouracil; TKI, tyrosine kinase inhibitor; MGMT, O6-methylguanine-DNA methyltransferase. Outcomes across rows are descriptive and should not be directly compared: treatment line, prior mitotane exposure, study design, sample size, and disease characteristics differ. EDP-M has randomized phase III evidence; most other encouraging data are from single-arm or retrospective studies.

## 8. Conclusions

The optimal sequencing of systemic therapies in advanced ACC remains uncertain and should be individualized according to tumor burden, rate of progression, hormonal activity, symptoms, prior treatment, performance status, and expert multidisciplinary consensus. EDP-M as the first-line regimen for patients with advanced ACC is supported by randomized phase III data and is generally preferred for patients with symptomatic, bulky, or rapidly progressive disease. Deferring EDP-M and using mitotane monotherapy as a first-line treatment may be considered for carefully selected patients with low-volume, indolent disease, although this strategy is supported mainly by observational evidence. After progression, cabozantinib, immunotherapy, temozolomide, and gemcitabine/fluoropyrimidine regimens have shown variable activity in studies with differing populations and designs. Small studies of combinations involving ICIs, TKIs, mitotane, or cisplatin provide signals for further investigation but do not establish these regimens as standards of care. Given the limited effectiveness of available therapies and the biological heterogeneity of ACC, clinical trial enrollment should be considered whenever feasible. Prospective trials and correlative studies are needed to clarify treatment sequencing, validate predictive biomarkers, and identify patients most likely to benefit from emerging treatments.

## Data Availability

No new data were created or analyzed in this study. Data sharing is not applicable to this article.

## References

[1] National Cancer Institute (1975). Third National Cancer Survey: Incidental Data.

[2] Sharma E., Dahal S., Sharma P., Bhandari A., Gupta V., Amgai B., Dahal S. (2018). The Characteristics and Trends in Adrenocortical Carcinoma: A United States Population Based Study. J. Clin. Med. Res..

[3] Puglisi S., Calabrese A., Ferrau F., Violi M.A., Lagana M., Grisanti S., Ceccato F., Scaroni C., Di Dalmazi G., Stigliano A. (2023). New Findings on Presentation and Outcome of Patients with Adrenocortical Cancer: Results from a National Cohort Study. J. Clin. Endocrinol. Metab..

[4] Gicquel C., Raffin-Sanson M.L., Gaston V., Bertagna X., Plouin P.F., Schlumberger M., Louvel A., Luton J.P., Le Bouc Y. (1997). Structural and functional abnormalities at 11p15 are associated with the malignant phenotype in sporadic adrenocortical tumors: Study on a series of 82 tumors. J. Clin. Endocrinol. Metab..

[5] Ragazzon B., Libe R., Gaujoux S., Assie G., Fratticci A., Launay P., Clauser E., Bertagna X., Tissier F., de Reynies A. (2010). Transcriptome analysis reveals that p53 and beta-catenin alterations occur in a group of aggressive adrenocortical cancers. Cancer Res..

[6] Juhlin C.C., Goh G., Healy J.M., Fonseca A.L., Scholl U.I., Stenman A., Kunstman J.W., Brown T.C., Overton J.D., Mane S.M. (2015). Whole-exome sequencing characterizes the landscape of somatic mutations and copy number alterations in adrenocortical carcinoma. J. Clin. Endocrinol. Metab..

[7] Assie G., Letouze E., Fassnacht M., Jouinot A., Luscap W., Barreau O., Omeiri H., Rodriguez S., Perlemoine K., Rene-Corail F. (2014). Integrated genomic characterization of adrenocortical carcinoma. Nat. Genet..

[8] Landwehr L.S., Altieri B., Schreiner J., Sbiera I., Weigand I., Kroiss M., Fassnacht M., Sbiera S. (2020). Interplay between glucocorticoids and tumor-infiltrating lymphocytes on the prognosis of adrenocortical carcinoma. J. Immunother. Cancer.

[9] Fay A.P., Signoretti S., Callea M., Telomicron G.H., McKay R.R., Song J., Carvo I., Lampron M.E., Kaymakcalan M.D., Poli-de-Figueiredo C.E. (2015). Programmed death ligand-1 expression in adrenocortical carcinoma: An exploratory biomarker study. J. Immunother. Cancer.

[10] Tierney J.F., Vogle A., Poirier J., Min I.M., Finnerty B., Zarnegar R., Pappas S.G., Scognamiglio T., Ghai R., Gattuso P. (2019). Expression of programmed death ligand 1 and 2 in adrenocortical cancer tissues: An exploratory study. Surgery.

[11] Raj N., Zheng Y., Kelly V., Katz S.S., Chou J., Do R.K.G., Capanu M., Zamarin D., Saltz L.B., Ariyan C.E. (2020). PD-1 Blockade in Advanced Adrenocortical Carcinoma. J. Clin. Oncol..

[12] Altieri B., Kircher S., Herterich S., Jahn A., Teleanu M.V., Lippert J., Landwehr L.S., Kimpel O., Reuter M., Remde H. (2026). Mismatch repair deficiency and microsatellite instability in adrenocortical carcinoma. ESMO Open.

[13] Bilimoria K.Y., Shen W.T., Elaraj D., Bentrem D.J., Winchester D.J., Kebebew E., Sturgeon C. (2008). Adrenocortical carcinoma in the United States: Treatment utilization and prognostic factors. Cancer.

[14] Daher M., Varghese J., Gruschkus S.K., Jimenez C., Waguespack S.G., Bedrose S., Altameemi L., Bazerbashi H., Naing A., Subaiah V. (2022). Temporal Trends in Outcomes in Patients with Adrenocortical Carcinoma: A Multidisciplinary Referral-center Experience. J. Clin. Endocrinol. Metab..

[15] Steenaard R.V., de Wit J.S., Rutjens M., van der Bruggen B., Blijdorp K., Wouters-van Poppel P., Feelders R.A., Haak H.R. (2026). Time trends in incidence, treatment and survival of patients with adrenocortical carcinoma, a nationwide study. J. Clin. Endocrinol. Metab..

[16] Puglisi S., Calabrese A., Basile V., Pia A., Reimondo G., Perotti P., Terzolo M. (2020). New perspectives for mitotane treatment of adrenocortical carcinoma. Best. Pract. Res. Clin. Endocrinol. Metab..

[17] Megerle F., Herrmann W., Schloetelburg W., Ronchi C.L., Pulzer A., Quinkler M., Beuschlein F., Hahner S., Kroiss M., Fassnacht M. (2018). Mitotane Monotherapy in Patients with Advanced Adrenocortical Carcinoma. J. Clin. Endocrinol. Metab..

[18] Mangone A., Altieri B., Ferrante E., Bancos I., Luconi M., Ziolkowska B., Nekic A.B., Libe R., Ceccato F., Pittaway J.F.H. (2026). Clinical predictors of outcome in advanced adrenocortical carcinoma: A multicenter international ENSAT study. Eur. J. Endocrinol..

[19] Debets P., Dreijerink K.M.A., Engelsman A., Dahele M., Haak H.R., Steenaard R.V., Kapiteijn E., Corssmit E., Menke-van der Houven van Oordt C.W. (2024). Impact of EDP-M on survival of patients with metastatic adrenocortical carcinoma: A population-based study. Eur. J. Cancer.

[20] Fassnacht M., Terzolo M., Allolio B., Baudin E., Haak H., Berruti A., Welin S., Schade-Brittinger C., Lacroix A., Jarzab B. (2012). Combination chemotherapy in advanced adrenocortical carcinoma. N. Engl. J. Med..

[21] Fassnacht M., Puglisi S., Kimpel O., Terzolo M. (2025). Adrenocortical carcinoma: A practical guide for clinicians. Lancet Diabetes Endocrinol..

[22] Berruti A., Terzolo M., Sperone P., Pia A., Della Casa S., Gross D.J., Carnaghi C., Casali P., Porpiglia F., Mantero F. (2005). Etoposide, doxorubicin and cisplatin plus mitotane in the treatment of advanced adrenocortical carcinoma: A large prospective phase II trial. Endocr. Relat. Cancer.

[23] Khan T.S., Imam H., Juhlin C., Skogseid B., Grondal S., Tibblin S., Wilander E., Oberg K., Eriksson B. (2000). Streptozocin and o,p′DDD in the treatment of adrenocortical cancer patients: Long-term survival in its adjuvant use. Ann. Oncol..

[24] Sperone P., Ferrero A., Daffara F., Priola A., Zaggia B., Volante M., Santini D., Vincenzi B., Badalamenti G., Intrivici C. (2010). Gemcitabine plus metronomic 5-fluorouracil or capecitabine as a second-/third-line chemotherapy in advanced adrenocortical carcinoma: A multicenter phase II study. Endocr. Relat. Cancer.

[25] Henning J.E.K., Deutschbein T., Altieri B., Steinhauer S., Kircher S., Sbiera S., Wild V., Schlotelburg W., Kroiss M., Perotti P. (2017). Gemcitabine-Based Chemotherapy in Adrenocortical Carcinoma: A Multicenter Study of Efficacy and Predictive Factors. J. Clin. Endocrinol. Metab..

[26] Grisanti S., Cosentini D., Lagana M., Morandi A., Lazzari B., Ferrari L., Volta A.D., Ambrosini R., Ferrari V.D., Sigala S. (2021). Clinical Prognostic Factors in Patients with Metastatic Adrenocortical Carcinoma Treated with Second Line Gemcitabine Plus Capecitabine Chemotherapy. Front. Endocrinol..

[27] Cosentini D., Badalamenti G., Grisanti S., Basile V., Rapa I., Cerri S., Spallanzani A., Perotti P., Musso E., Lagana M. (2019). Activity and safety of temozolomide in advanced adrenocortical carcinoma patients. Eur. J. Endocrinol..

[28] Zhulikov Y.A., Kovalenko E.I., Bohian V., Evdokimova E.V. (2026). 65P Temozolomide as second and subsequent lines of treatment in metastatic adrenocortical cancer. Single-center retrospective trial. ESMO Rare Cancers.

[29] Lagana M., Grisanti S., Ambrosini R., Cosentini D., Abate A., Zamparini M., Ferrari V.D., Gianoncelli A., Turla A., Canu L. (2022). Phase II study of cabazitaxel as second-third line treatment in patients with metastatic adrenocortical carcinoma. ESMO Open.

[30] Catalano R., Nozza E., Altieri B., Esposito E., Croci G.A., Barbieri A.M., Treppiedi D., Di Bari S., Kimpel O., Detomas M. (2025). Emerging role of IGF1R and IR expression and localisation in adrenocortical carcinomas. Cell Commun. Signal..

[31] Fassnacht M., Berruti A., Baudin E., Demeure M.J., Gilbert J., Haak H., Kroiss M., Quinn D.I., Hesseltine E., Ronchi C.L. (2015). Linsitinib (OSI-906) versus placebo for patients with locally advanced or metastatic adrenocortical carcinoma: A double-blind, randomised, phase 3 study. Lancet Oncol..

[32] Naing A., Lorusso P., Fu S., Hong D., Chen H.X., Doyle L.A., Phan A.T., Habra M.A., Kurzrock R. (2013). Insulin growth factor receptor (IGF-1R) antibody cixutumumab combined with the mTOR inhibitor temsirolimus in patients with metastatic adrenocortical carcinoma. Br. J. Cancer.

[33] Xu Y.Z., Zhu Y., Shen Z.J., Sheng J.Y., He H.C., Ma G., Qi Y.C., Zhao J.P., Wu Y.X., Rui W.B. (2011). Significance of heparanase-1 and vascular endothelial growth factor in adrenocortical carcinoma angiogenesis: Potential for therapy. Endocrine.

[34] Kroiss M., Megerle F., Kurlbaum M., Zimmermann S., Wendler J., Jimenez C., Lapa C., Quinkler M., Scherf-Clavel O., Habra M.A. (2020). Objective Response and Prolonged Disease Control of Advanced Adrenocortical Carcinoma with Cabozantinib. J. Clin. Endocrinol. Metab..

[35] Campbell M.T., Balderrama-Brondani V., Jimenez C., Tamsen G., Marcal L.P., Varghese J., Shah A.Y., Long J.P., Zhang M., Ochieng J. (2024). Cabozantinib monotherapy for advanced adrenocortical carcinoma: A single-arm, phase 2 trial. Lancet Oncol..

[36] Kroiss M., Quinkler M., Johanssen S., van Erp N.P., Lankheet N., Pollinger A., Laubner K., Strasburger C.J., Hahner S., Muller H.H. (2012). Sunitinib in refractory adrenocortical carcinoma: A phase II, single-arm, open-label trial. J. Clin. Endocrinol. Metab..

[37] van Erp N.P., Guchelaar H.J., Ploeger B.A., Romijn J.A., Hartigh J., Gelderblom H. (2011). Mitotane has a strong and a durable inducing effect on CYP3A4 activity. Eur. J. Endocrinol..

[38] O’Sullivan C., Edgerly M., Velarde M., Wilkerson J., Venkatesan A.M., Pittaluga S., Yang S.X., Nguyen D., Balasubramaniam S., Fojo T. (2014). The VEGF inhibitor axitinib has limited effectiveness as a therapy for adrenocortical cancer. J. Clin. Endocrinol. Metab..

[39] Flauto F., Damiano V. (2025). The Efficacy and Safety of Multi-Kinase Inhibitors in Adrenocortical Carcinoma: A Systematic Review and Single-Arm Meta-Analysis. Cancers.

[40] Chahla B., Stephen B., Song J., Balderrama-Brondani V., Yaylaci F., Campbell M.T., Naing A., Habra M.A. (2025). Phase 2 Study of Monotherapy with Pembrolizumab for Advanced Adrenocortical Carcinoma. J. Immunother. Precis. Oncol..

[41] Carneiro B.A., Konda B., Costa R.B., Costa R.L.B., Sagar V., Gursel D.B., Kirschner L.S., Chae Y.K., Abdulkadir S.A., Rademaker A. (2019). Nivolumab in Metastatic Adrenocortical Carcinoma: Results of a Phase 2 Trial. J. Clin. Endocrinol. Metab..

[42] Le Tourneau C., Hoimes C., Zarwan C., Wong D.J., Bauer S., Claus R., Wermke M., Hariharan S., von Heydebreck A., Kasturi V. (2018). Avelumab in patients with previously treated metastatic adrenocortical carcinoma: Phase 1b results from the JAVELIN solid tumor trial. J. Immunother. Cancer.

[43] Patel S.P., Othus M., Chae Y.K., Huynh T., Tan B., Kuzel T., McLeod C., Lopez G., Chen H.X., Sharon E. (2024). Phase II basket trial of Dual Anti-CTLA-4 and Anti-PD-1 blockade in Rare Tumors (DART) SWOG S1609: Adrenocortical carcinoma cohort. J. Immunother. Cancer.

[44] Remde H., Schmidt-Pennington L., Reuter M., Landwehr L.S., Jensen M., Lahner H., Kimpel O., Altieri B., Laubner K., Schreiner J. (2023). Outcome of immunotherapy in adrenocortical carcinoma: A retrospective cohort study. Eur. J. Endocrinol..

[45] Giner I.S., Resende J.S.S., Muzzi J.C.D., Barbuto J.A.M., Lalli E., Castro M.A.A., Figueiredo B.C. (2026). Steroid Phenotype Stratification Reveals Distinct HLA Expression Signatures in Adrenocortical Carcinoma. Cancers.

[46] Bedrose S., Miller K.C., Altameemi L., Ali M.S., Nassar S., Garg N., Daher M., Eaton K.D., Yorio J.T., Daniel D.B. (2020). Combined lenvatinib and pembrolizumab as salvage therapy in advanced adrenal cortical carcinoma. J. Immunother. Cancer.

[47] Park I., Kwon M., Park K., Park S.H., Park J.-H., Shin S.J., Shim H.-J., Yun T., Lee J.-L., Lee H.J. (2023). A prospective phase II trial of pembrolizumab plus lenvatinib in advanced adrenal cortical carcinoma after failure of platinum- and mitotane-based chemotherapy (ACCOMPLISH). J. Clin. Oncol..

[48] Zhu Y.C., Wei Z.G., Wang J.J., Pei Y.Y., Jin J., Li D., Li Z.H., Liu Z.R., Min Y., Li R.D. (2024). Camrelizumab plus apatinib for previously treated advanced adrenocortical carcinoma: A single-arm phase 2 trial. Nat. Commun..

[49] Capdevila J., Hernando J., Molina-Cerrillo J., Benavent Vinuales M., Garcia-Carbonero R., Teule A., Custodio A., Jimenez-Fonseca P., Lopez C., Hierro C. (2025). Cabozantinib plus Atezolizumab in Advanced, Progressive Endocrine Malignancies: A Multicohort, Basket, Phase II Trial (CABATEN/GETNE-T1914). Clin. Cancer Res..

[50] Lagana M., Rodella S., Bettini D.L., Esposito A., Abate A., Ambrosini R., Tamburello M., Consoli F., Tinti R., Calza S. (2026). Chemo-immunotherapy with cisplatin and nivolumab as second-line approach in metastatic adrenocortical carcinoma. Eur. J. Endocrinol..

[51] Head L., Kiseljak-Vassiliades K., Clark T.J., Somerset H., King J., Raeburn C., Albuja-Cruz M., Weyant M., Cleveland J., Wierman M.E. (2019). Response to Immunotherapy in Combination with Mitotane in Patients with Metastatic Adrenocortical Cancer. J. Endocr. Soc..

[52] Del Rivero J., Glod J., Magee T., Cooper K., Rivero A., Lanfranconi T., Patel R., Figg W.D., Pommier Y., Wide-mann B.C. (2025). A first-in-human phase I trial with antibody drug conjugate ADCT-701 in neuroendocrine tumors and carcinomas. J. Clin. Oncol..

[53] Sun N.Y., Kumar S., Kim Y.S., Varghese D., Mendoza A., Nguyen R., Okada R., Reilly K., Widemann B., Pommier Y. (2025). Identification of the Notch ligand DLK1 as an immunotherapeutic target and regulator of tumor cell plasticity and chemoresistance in adrenocortical carcinoma. Nat. Commun..

[54] Hahner S., Hartrampf P.E., Mihatsch P.W., Nauerz M., Heinze B., Hanscheid H., Teresa Fuss C., Werner R.A., Pamporaki C., Kroiss M. (2022). Targeting 11-Beta Hydroxylase with [131I]IMAZA: A Novel Approach for the Treatment of Advanced Adrenocortical Carcinoma. J. Clin. Endocrinol. Metab..

[55] Michalski K., Schlotelburg W., Hartrampf P.E., Kosmala A., Buck A.K., Hahner S., Schirbel A. (2023). Radiopharmaceuticals for Treatment of Adrenocortical Carcinoma. Pharmaceuticals.

[56] Hescot S., Debien V., Hadoux J., Drui D., Haissaguerre M., de la Fouchardiere C., Vezzosi D., Do Cao C., Libe R., Le Tourneau C. (2023). Outcome of adrenocortical carcinoma patients included in early phase clinical trials: Results from the French network ENDOCAN-COMETE. Eur. J. Cancer.

